# Childhood psychosocial adversity and female reproductive timing: a cohort study of the ALSPAC mothers

**DOI:** 10.1136/jech-2017-209488

**Published:** 2017-11-09

**Authors:** Maria C Magnus, Emma L Anderson, Laura D Howe, Carol J Joinson, Ian S Penton-Voak, Abigail Fraser

**Affiliations:** 1 MRC Integrative Epidemiology Unit, University of Bristol, Bristol, UK; 2 Department of Population Health Sciences, Bristol Medical School, Bristol, UK; 3 Division for Mental and Physical Health, Norwegian Institute of Public Health, Oslo, Norway; 4 NIHR Bristol Biomedical Research Centre, University Hospitals Bristol NHS Foundation Trust and University of Bristol, Bristol, UK; 5 School of Experimental Psychology, University of Bristol, Bristol, UK

**Keywords:** psychosocial Factors, reproductive health, epidemiology

## Abstract

**Background:**

Previous studies of childhood psychosocial adversity and age at menarche mostly evaluated single or a few measures of adversity, and therefore could not quantify total psychosocial adversity. Limited knowledge is currently available regarding childhood psychosocial adversity in relation to age at menopause and reproductive lifespan.

**Methods:**

We examined the associations of total and specific components of childhood psychosocial adversity with age at menarche (n=8984), age at menopause (n=945), and length of reproductive lifespan (n=841), in mothers participating in the Avon Longitudinal Study of Parents and Children. We used confirmatory factor analysis to characterise lack of care, maladaptive family functioning, non-sexual abuse, overprotective parenting, parental mental illness and sexual abuse. These specific components of childhood psychosocial adversity were combined into a total psychosocial adversity score using a second-order factor analysis. We used structural equation models to simultaneously conduct the factor analysis and estimate the association with the continuous outcomes of interest.

**Results:**

Total childhood psychosocial adversity was not associated with age at menarche, age at menopause or length of reproductive lifespan. When we examined the separate psychosocial adversity constructs, sexual abuse was inversely associated with age at menarche, with a mean difference of −0.17 (95% CI −0.23 to −0.12) years per SD higher factor score, and with age at menopause, with a mean difference of −0.17 (95% CI −0.52 to 0.18) per SD higher factor score.

**Conclusion:**

Childhood sexual abuse was associated with lower age at menarche and menopause, but the latter needs to be confirmed in larger samples.

## Introduction

Psychosocial adversity in childhood/adolescence can have a broad influence on development, including reproductive timing.^1^ Different aspects of childhood psychosocial adversity have been explored in relation to age at menarche, including paternal absence,^2–9^ parental divorce,^5 10 11^ parenting/family characteristics,^2 4 7 10 12 13^ parental marital satisfaction,^11^ parental mental illness,^4 5 12^ placement in foster care/adoption,^2 14^ death of a parent,^5 13^ and physical or sexual abuse.^2 5 14–20^ This literature suggests that greater childhood psychosocial adversity is associated with earlier sexual maturity.

Most of these previous studies, although not all,^2 5^ evaluated a single or a small number of measures of psychosocial adversity in relation to age at menarche, and were therefore limited in their ability to capture total childhood psychosocial adversity. This is important because exposure to multiple adverse experiences in childhood is more detrimental than experiencing only one. Less is known about childhood psychosocial adversity in relation to reproductive health later in life. One study reported that women who experience parental divorce during childhood have earlier menopause, while another found that women who experience physical or sexual abuse in childhood experience menopause later.^21 22^

Several theories are proposed for how childhood psychosocial adversity can influence reproductive timing. The psychosocial acceleration theory states that childhood adversity accelerates sexual maturity via stress hormones activating the hypothalamic–pituitary–gonadal axis prematurely.^23^ Experimental animal studies also show that increased levels of stress hormones might accelerate ovarian follicular depletion,^24^ resulting in earlier menopause. It is therefore plausible that greater levels of stress hormones might be a common explanation for how childhood psychosocial adversity might accelerate both age at menarche and age at menopause. Additional theories are also proposed for how childhood psychosocial adversity could be associated with age at menarche. The child development theory describes how the composition and quality of family environments might influence age at menarche as a developmental strategy, while the parental investment theory postulates a more specific role of biological father absence and/or stepfather presence in female pubertal timing, but the potential biological mechanisms remain unclear.^25 26^ Finally, in contrast to the psychosocial acceleration theory, the stress suppression theory suggests that childhood adversity results in a later age at menarche, as a way of delaying reproduction until better circumstances are achieved.^27^

The objective of the current study was therefore to examine associations of total childhood psychosocial adversity with age at menarche, age at menopause and length of the reproductive lifespan in mothers from a large contemporary population-based British pregnancy cohort. Using a wide range of measures of childhood psychosocial adversity, we also set out to evaluate whether certain components of adversity might be more closely associated with female reproductive timing than others, to further clarify potential explanatory mechanisms.

## Materials and methods

### The Avon Longitudinal Study of Parents and Children

The Avon Longitudinal Study of Parents and Children (ALSPAC) recruited women with expected delivery dates between April 1991 and December 1992 living in a defined area of Avon, South West England.^28 29^ The participation rate of invited pregnant women was 75.3%, resulting in 14 541 participating women. Written informed consent was obtained from all participants. A fully searchable data dictionary is available online.^30^

### Childhood psychosocial adversity

Mothers from the ALSPAC cohort (with mean age of 28 years at recruitment; SD 5 years) were asked to retrospectively recall elements related to their childhood psychosocial adversity in questionnaires administered at approximately 12 gestational weeks (at recruitment), at 32 gestational weeks and when the offspring was about 3 years (approximately 4 years after recruitment). The questions asked mothers to report perceived maternal lack of care (12 questions), maternal overprotection (7 questions), maladaptive family functioning (12 questions), parental mental illness (4 questions), sexual abuse (5 questions) and non-sexual abuse (4 questions). Further information on the specific questions under each of these categories is given in online supplementary table 1.

10.1136/jech-2017-209488.supp1 Supplementary file 1

### Ages at menarche and natural menopause, and length of reproductive lifespan

Participants reported their age at menarche retrospectively in whole years at the time of recruitment. Age at natural menopause was also self-reported. We combined information from a questionnaire administered approximately 19 years after recruitment (at a mean age of 47 years) with information from two clinic visits when participants were a mean of 48 and 51 years, respectively. Participants were asked (1) whether they had a period or menstrual bleeding in the past 12 months and (2) when they experienced their last menstrual period. If participants had not had a menstrual period in the past 12 months, they indicated whether this was due to (1) surgery; (2) chemotherapy or radiation therapy; (3) no obvious reason/menopause; (4) other reasons including, for example, pregnancy/breast feeding or use of contraceptives. We used the answer option ‘no obvious reason/menopause’ to indicate natural menopause. Length of reproductive lifespan was calculated as age of menopause minus the age at menarche.

### Covariates

The ALSPAC mothers reported their age in years, ethnicity (white/European vs other) and educational qualifications (A level or above/O level or below) at baseline. Information regarding their father’s occupation (manual vs non-manual) and parental highest educational level (A level or above/O level or below), indicating childhood socioeconomic position, was available in approximately 70% of the study sample.

### Statistical analysis

**Figure 1:**
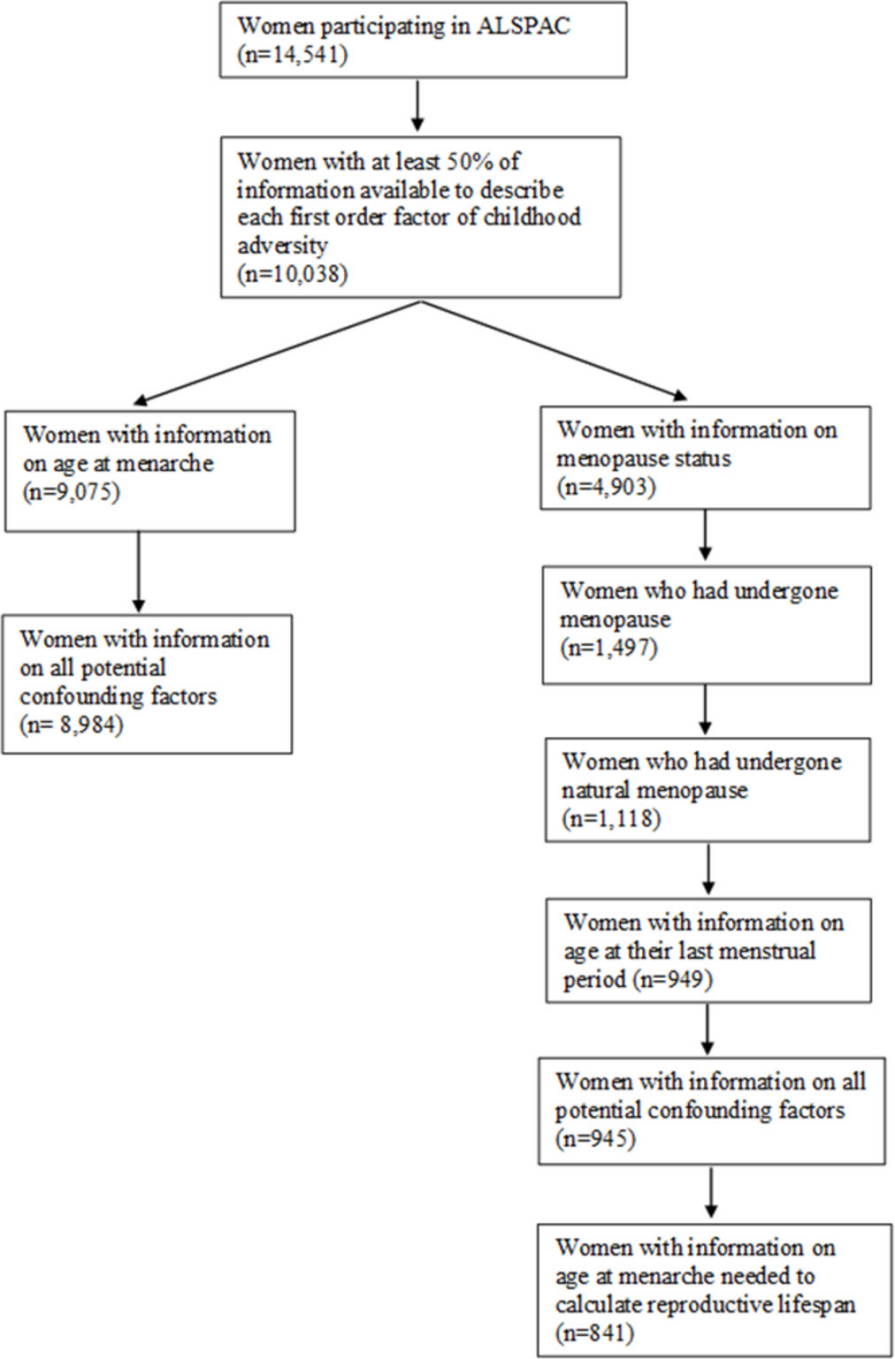
Illustration of sample. ALSPAC, Avon Longitudinal Study of Parents and Children.

We sought to combine all questions available for each form of psychosocial adversity into a single variable. Therefore, we used confirmatory factor analysis to create single latent constructs for maternal lack of care, maternal overprotection, maladaptive family functioning, parental mental illness, sexual abuse and non-sexual abuse (online supplementary figure 1). The confirmatory factor analysis uses information on the correlation structure of a set of observed variables to inform a single a priori hypothesised latent construct. For example, we used information on the answer to 12 questions regarding characteristics of the parents’ relationship, parental separation/divorce and paternal absence to define the underlying latent construct which we called maladaptive family functioning. Higher values for the latent construct identified in the first-order factor analysis reflect higher levels of childhood psychosocial adversity. These six latent constructs from the first-order confirmatory factor analysis were then used to inform a single latent construct reflecting the total childhood psychosocial adversity in a second-order factor analysis. Women with at least 50% of covariates used to inform each of the six latent constructs of childhood psychosocial adversity were eligible for the analysis (n=10 038), to ensure that the definition of the factors was not driven by single measures with the least amount of missing information (figure 1).

The factor loadings for the observed variables onto the six latent constructs in the first-order factor analysis, in addition to the factor loadings of the six latent constructs onto the single latent construct of the second-order factor analysis, are displayed in online supplementary table 1. Measures of childhood psychosocial adversity that are strongly correlated with other measures of childhood psychosocial adversity, and therefore more likely to co-occur, have higher factor loadings. We examined the model fit for the six first-order factor analyses and the single second-order factor analysis using root mean square error of approximations, comparative fit index and Tucker-Lewis fit index, which are displayed in online supplementary table 1. The model fit statistics for this confirmatory factor analysis were reasonable.

Structural equation models (SEM) were used to simultaneously conduct the factor analyses of childhood psychosocial adversity as described above and examine their associations with the continuous outcomes of interest. The effect estimates indicate the mean difference in the outcome per SD increase in the latent construct reflecting childhood psychosocial adversity. We constructed an unadjusted model (model 1), a model adjusted for age at recruitment and ethnicity (model 2), and a model further adjusting adult educational qualifications (model 3). We adjusted for adult educational qualifications in a separate model, since it could plausibly be on the causal pathway, particularly for age at menopause and length of reproductive lifespan. A sensitivity analysis adjusted for parental highest educational level and paternal occupation as direct measures of childhood socioeconomic position in the subsample with this information available. The SEM dealt with missing data using a weighted least squares mean and variance adjusted estimator, which assumes missing at random. We subsequently repeated the analysis restricted to individuals with information on all variables used to inform the six latent constructs in the first-order factor analysis. We also conducted a sensitivity analysis including all individuals with information on at least one of the measures of psychosocial adversity used to inform the latent constructs in the first-order factor analysis. Finally, we investigated the associations of paternal absence with the outcomes of interests more closely using linear regression.

The analyses were conducted on Mplus V.7.31 (Muthén & Muthén, 2008) and Stata V.14.

## Results

Of the 10 038 eligible women, 8984 with information on childhood psychosocial adversity, covariates and age at menarche were included in the analysis of age at menarche (figure 1). Those with the information necessary to be included in the primary analysis were older, more likely to be European/white and more likely to have higher educational attainment than those without sufficient information (online supplementary table 2). A total of 4903 women had information on menopausal status, out of whom 945 had experienced natural menopause and had information on their age at the last menstrual period (figure 1). Eight hundred and forty-one were included in the analysis of length of reproductive lifespan (figure 1). The women included in the secondary analysis (age at natural menopause and length of reproductive lifespan) were older and on average had a higher educational level compared with those included in the primary analysis (age at menarche) (table 1). There was a modest to strong correlation between the six factors identified in the first-order factor analysis (online supplementary table  3).

**Table 1:** Distribution of background characteristics in each analysis sample

Characteristics	Age at menarche in years (n=8984)	Age at menopause in years (n=945)	Length of reproductive lifespan in years (n=841)
Age at delivery/enrolment	28.5 (4.8)	34.2 (3.4)	34.2 (3.4)
Ethnicity			
White/European	8798 (97.9)	927 (98.1)	823 (97.9)
Other	186 (2.1)	18 (1.9)	18 (2.1)
Educational qualifications			
A level or above	3499 (39.0)	555 (58.7)	495 (58.9)
O level or below	5485 (61.0)	390 (41.3)	346 (41.1)
Parental highest educational qualifications			
A level or above	1784 (19.9)	232 (24.6)	210 (25.0)
O level or below	4666 (51.9)	489 (51.7)	432 (51.4)
Missing	2534 (28.2)	224 (23.7)	199 (23.7)
Paternal occupation			
Non-manual	3505 (39.0)	487 (51.5)	429 (51.0)
Manual	3806 (42.3)	330 (35.0)	296 (35.2)
Missing	1673 (18.6)	128 (13.5)	116 (13.8)

### Childhood psychosocial adversity and age at menarche

The mean age at menarche was 12.9 years (SD: 1.5; range 8–19 years). We observed no strong evidence of an association between total childhood psychosocial adversity and age at menarche (table 2). When we examined associations of different types of psychosocial adversity with age at menarche, we observed an inverse association between the latent construct for sexual abuse and age at menarche in both unadjusted and confounder-adjusted models (table 2). There was no strong evidence of associations with any other types of childhood psychosocial adversity (maternal lack of care, maladaptive family functioning, non-sexual abuse, maternal overprotective parenting and parental mental illness) (table 2).

**Table 2:** Associations of childhood psychosocial adversity with age at menarche (n=8984)

Exposure	Model 1		Model 2		Model 3	
	Mean difference in years (95% CI)	P value	Mean difference in years (95% CI)	P value	Mean difference in years (95% CI)	P value
Total psychosocial adversity	0.005 (−0.034 to 0.044)	0.790	0.007 (−0.032 to 0.046)	0.729	0.006 (−0.033 to 0.045)	0.778
Lack of care	0.027 (−0.008 to 0.062)	0.134	0.023 (−0.012 to 0.058)	0.198	0.022 (−0.013 to 0.057)	0.223
Maladaptive family functioning	0.013 (−0.026 to 0.052)	0.527	0.023 (−0.016 to 0.062)	0.251	0.022 (−0.017 to 0.061)	0.273
Non-sexual abuse	−0.007 (−0.056 to 0.042)	0.777	−0.003 (−0.052 to 0.046)	0.897	−0.002 (−0.051 to 0.047)	0.943
Overprotective parenting	−0.003 (−0.040 to 0.034)	0.881	−0.008 (−0.045 to 0.029)	0.671	−0.009 (−0.046 to 0.028)	0.631
Parental mental illness	−0.027 (−0.076 to 0.022)	0.280	−0.030 (−0.079 to 0.019)	0.220	−0.030 (−0.079 to 0.019)	0.220
Sexual abuse	−0.182 (−0.235 to 0.129)	<0.001	−0.172 (−0.225 to 0.119)	<0.001	−0.173 (−0.226 to 0.120)	<0.001

Beta coefficients are interpreted as a mean difference in age at menarche in years per SD increase in psychosocial adversity.

Model 1: unadjusted.

Model 2: adjusted for age at recruitment and ethnicity.

Model 3: adjusted for age at recruitment, educational qualifications and ethnicity.

### Childhood psychosocial adversity and age at menopause and reproductive lifespan

The mean age at menopause was 48.6 years (SD 3.8; range 35–58 years), while the mean length of reproductive lifespan was 35.7 years (SD 4.0; range 21–45 years). There was no strong evidence of associations of total childhood psychosocial adversity, nor any of the different types of childhood psychosocial adversity, with age at menopause (table 3) or length of reproductive lifespan (table 4).

**Table 3:** Associations of childhood psychosocial adversity with age at menopause (n=945)

Exposure	Model 1		Model 2		Model 3	
	Mean difference in years (95% CI)	P value	Mean difference in years (95% CI)	P value	Mean difference in years (95% CI)	P value
Total psychosocial adversity	0.000 (−0.290 to 0.290)	0.998	−0.010 (−0.275 to 0.255)	0.940	−0.023 (−0.286 to 0.240)	0.864
Lack of care	0.067 (−0.205 to 0.339)	0.631	0.047 (−0.198 to 0.292)	0.706	0.036 (−0.207 to 0.279)	0.770
Maladaptive family functioning	−0.103 (−0.395 to 0.189)	0.487	−0.024 (−0.287 to 0.239)	0.856	−0.021 (−0.286 to 0.244)	0.877
Non-sexual abuse	0.018 (−0.364 to 0.400)	0.927	0.004 (−0.341 to 0.349)	0.982	−0.014 (−0.343 to 0.315)	0.934
Overprotective parenting	0.061 (−0.223 to 0.345)	0.674	−0.076 (−0.339 to 0.187)	0.569	−0.090 (−0.351 to 0.171)	0.498
Parental mental illness	−0.229 (−0.613 to 0.155)	0.244	−0.111 (0.444 to 0.222)	0.514	−0.154 (−0.489 to 0.181)	0.368
Sexual abuse	−0.189 (−0.575 to 0.197)	0.338	−0.169 (−0.518 to 0.180)	0.342	−0.182 (−0.529 to 0.165)	0.304

Beta coefficients are interpreted as the mean difference in age at menopause in years per SD increase in psychosocial adversity.

Model 1: unadjusted.

Model 2: adjusted for age at recruitment and ethnicity.

Model 3: adjusted for age at recruitment, educational qualifications and ethnicity.

**Table 4:** Associations of childhood psychosocial adversity with length of reproductive lifespan (n=841)

Exposure	Model 1		Model 2		Model 3	
	Mean difference in years (95% CI)	P value	Mean difference in years (95% CI)	P value	Mean difference in years (95% CI)	P value
Total psychosocial adversity	−0.016 (−0.337 to 0.305)	0.922	−0.024 (−0.318 to 0.270)	0.873	−0.040 (−0.331 to 0.250)	0.786
Lack of care	−0.008 (−0.308 to 0.292)	0.957	−0.040 (−0.309 to 0.229)	0.770	−0.057 (−0.324 to 0.210)	0.673
Maladaptive family functioning	0.001 (−0.322 to 0.324)	0.993	0.114 (−0.180 to 0.408)	0.448	0.122 (−0.172 to 0.416)	0.414
Non-sexual abuse	−0.025 (−0.425 to 0.375)	0.901	−0.052 (−0.415 to 0.311)	0.779	−0.057 (−0.406 to 0.292)	0.748
Overprotective parenting	0.028 (−0.291 to 0.347)	0.864	−0.142 (−0.440 to 0.156)	0.350	−0.169 (−0.465 to 0.127)	0.263
Parental mental illness	−0.082 (−0.517 to 0.353)	0.713	0.024 (−0.372 to 0.420)	0.905	−0.012 (−0.404 to 0.380)	0.951
Sexual abuse	−0.084 (−0.515 to 0.347)	0.701	−0.044 (−0.444 to 0.356)	0.830	−0.069 (−0.461 to 0.323)	0.729

Beta coefficients are interpreted as a mean difference in length of reproductive lifespan in years per SD increase in psychosocial adversity.

Model 1: unadjusted.

Model 2: adjusted for age at recruitment and ethnicity.

Model 3: adjusted for age at recruitment, educational qualifications and ethnicity.

### Sensitivity analyses

Multivariable adjustment for direct measures of childhood socioeconomic position did not change the associations (online supplementary tables 4–6). The analysis restricted to individuals with complete information on all variables used to inform the six latent constructs (online supplementary tables 7–9), or to individuals with at least one measure used to inform the six latent constructs (online supplementary tables 10–12), also yielded similar associations. When we restricted the analysis of age at menarche to the study sample for length of reproductive lifespan (n=841), the association with the latent construct for parental mental illness was strengthened, while the association with sexual abuse was attenuated (online supplementary table 13). Finally, paternal absence before the age of 5 was not associated with any of the outcomes after adjusting for potential confounders (online supplementary table 14). However, paternal absence first occurring between 6 and 11 years of age was associated with an earlier age at menopause and more weakly with shorter length of reproductive lifespan (online supplementary table 14). Notably, paternal absence was positively associated with the other five latent constructs from the first-order factor analysis (online supplementary table 15).

## Discussion

In this contemporary British cohort, total childhood psychosocial adversity was not associated with female reproductive timing. When we examined various components of childhood psychosocial adversity, childhood sexual abuse was associated with a younger age at menarche, and there was weak evidence of an association with younger age at menopause. Paternal absence, specifically paternal absence first occurring between 6 and 11 years of age, showed an association with earlier menopause and shorter reproductive lifespan.

### Strengths and limitations

The main strengths of our study is the large size and the range of measures of childhood psychosocial adversity. Since we evaluated multiple measures of childhood psychosocial adversity and three outcomes, we cannot exclude an influence of chance. The retrospectively reported measures of childhood psychosocial adversity were largely based on perceptions that could have been modified in the light of experience over time. We also could not establish with certainty whether the adversity had been experienced before or after menarche, as participants were asked about sexual abuse up to the age of 16/17 years. We relied on recall of age at menarche at a mean age of 28 years. There is mixed evidence of the ability of women to recall their age at menarche.^31 32^ Furthermore, age at menarche in whole years is a rather crude measure, which might make it difficult to capture more subtle differences. There was also a substantial loss to follow-up at the time points when we gathered information to define age at menopause. We therefore had lower statistical power for the secondary outcomes, and we cannot rule out an influence of selection. In addition, the overall proportion of women who had undergone natural menopause was relatively low in this cohort (22%). Of the 1497 women who had undergone menopause, 379 (25%) had undergone surgical menopause and 1118 (75%) had undergone natural menopause. This likely reflects the young age of the cohort, and the proportion of women with natural menopause will increase as the cohort ages. Notably, we did not observe similar associations of childhood psychosocial adversity with age at menarche in the subsample included in the analysis of length of reproductive lifespan, which could be explained by the age and/or socioeconomic differences among the two samples.

### Comparison with previous studies

Two previous studies quantified total childhood adversity in relation to age at menarche, using the absolute number of childhood adversities as the exposure.^2 5^ Results from the National Child Development Study that evaluated six childhood psychosocial adversity indicators found that a higher absolute number of childhood psychosocial adversities was associated with later age at menarche.^2^ However, consistent with our findings, sexual abuse was the indicator most strongly associated with early menarche, with an OR for menarche at 11 years or younger of 2.60 (95% CI 1.40 to 4.81) compared with those with menarche at 12 or 13 years of age.^2^ Findings from the National Comorbidity Survey-Replication including 11 different childhood psychosocial adversities indicated that 5 were associated with an increased risk of menarche at 11 years or younger, and that childhood sexual abuse was the only adversity associated with early menarche after adjustment for co-occurring adversities, with an OR of 1.77 (95% CI 1.21 to 2.60) compared with those with at menarche at 12 years or older.^5^

In contrast to previous studies, we observed no associations of non-sexual abuse,^2 5 14 15 18^ or parenting/family characteristics,^2 4 7 10 12 13^ with age at menarche. Notably, the type of information available on parenting/family characteristics varied greatly across studies.^2 4 7 10 12 13^ We also did not replicate the inverse association observed between paternal absence and age at menarche in previous studies.^2–9^

Less evidence is available on childhood psychosocial adversity and age at menopause.^21 22 33^ Analyses of 1515 women ages 47–53 years in the UK 1946 Birth Cohort indicated that women who experienced parental divorce early in life (before age 5 years) were more likely to be postmenopausal at the time of study (HR 2.14; 95% CI 1.33 to 3.42).^21^ In contrast to these results, we did not observe an association with paternal absence the first 5 years of life, but found that paternal absence first occurring between 6 and 11 years was associated with an earlier menopause. Results from the Harvard Study of Moods and Cycles further found that childhood physical and sexual abuse was associated with higher levels of both follicle stimulating hormone and oestradiol among premenopausal women 41–45 years of age, which could predict an earlier menopausal transition.^33^ Seemingly in contrast to these findings of premenopausal hormone levels, later findings from this cohort showed that women who had experienced childhood physical childhood abuse entered perimenopause at an older age.^22^

### Potential explanatory mechanisms

The heterogeneity in the reported associations between the different measures of childhood adversity and female reproductive timing across existing studies might reflect differences between cohorts over time and between geographical areas. This notion is supported by the fact that a previous study of paternal absence and age at menarche in the ALSPAC offspring, that is, including the daughters of women included in the current study, reported an earlier menarche among those who had experienced paternal absence.^9^

Our findings (and others^2 5^) suggest a specific association between sexual abuse and earlier menarche. We did not observe similar associations with the other types of childhood psychosocial adversity, nor with total childhood psychosocial adversity. We interpret this as indicating that the association reflects something unique to sexual abuse, particularly as these findings are consistent with previous reports. Childhood sexual abuse is likely to be the most severe psychosocial adversity that we captured. Notably, we could not clearly establish the temporal direction of the association, and there is literature indicating that women who experience menarche at an earlier age might be more vulnerable to childhood/adolescent sexual abuse.^34–36^

The observed link between stress hormones and accelerated depletion of the ovarian follicular could potentially be an explanation for the inverse association between paternal absence and age at menopause.^24^ Since women are born with a finite number of ovarian follicles, an accelerated depletion can result in an earlier menopause.^37^ While we acknowledge that sexual abuse is likely to be the most severe childhood experience we were able to capture, it is possible that paternal absence could be a stronger indicator of cumulative psychosocial adversity across the life course, which might in turn be more likely to influence age at menopause as opposed to age at menarche. For example, paternal absence is plausibly associated with a broad range of measures of social disadvantage and lifestyle characteristics that are difficult to capture, which could in turn be linked to higher levels of stress hormones across the life course and subsequent accelerated ovarian follicular depletion.^38 39^ Further information is necessary to substantiate this hypothesis.

## Conclusion

Childhood sexual abuse was associated with a lower age at menarche, and showed some evidence of an association with a lower age at menopause, but this needs to be examined in larger samples. Paternal absence was also associated with earlier age at menopause in secondary analyses. Other aspects of childhood psychosocial adversity showed no strong evidence of associations with female reproductive timing.

**What is already known on this subject**

- The majority of previous studies looking at childhood psychosocial adversity and age at menarche could not quantify total psychosocial adversity.
- Limited knowledge is currently available regarding whether any aspects of childhood psychosocial adversity might be associated with age at menopause or length of reproductive lifespan.
- It therefore remains unclear if any of the previously reported associations between childhood psychosocial adversity and earlier menarche might also be reflected in an earlier menopausal transition.

**What this study adds**

- In our comprehensive confirmatory factor analysis of childhood psychosocial adversity, we observed no association between total childhood psychosocial adversity and age at menarche, age at menopause or length of reproductive lifespan.
- Our findings inciated a robust association specific to childhood sexual abuse with earlier menarche.
- This is the first study to report that childhood sexual abuse might also be associated with earlier menopause, but this needs to be confirmed in larger studies.
