# Supplementary material for: Childhood psychosocial adversity and female reproductive timing: a cohort study of the ALSPAC mothers
Source: J Epidemiol Community Health. 2017 Nov 9;72(1):34–40. doi: 10.1136/jech-2017-209488 (PMC5753025; doi:10.1136/jech-2017-209488)

Table S1 Prevalence of measures used in first order factor analyses of childhood psychosocial adversity and model fit statistics

Factor	Question	Prevalence N (% exposed)	Factor loading	Model fit statistics
Sexual abuse	Did anyone masturbate in front of you before you were 16 without your consent?	8,841 (4.1)	0.757	RMSEA 0.037 CFI 0.993 TLI 0.986
	Did anyone have sexual intercourse with you before you were 16 without your consent?	8,984 (2.1)	0.889	
	Did anybody rub their genitals against your body in a sexual way before you were 16 without your consent?	8,984 (6.0)	0.924	
	Did anyone ever touch or fondle your body, including your breast or genitals, or attempt to arouse you sexually before you were 16 without your consent?	8,984 (10.2)	0.895	
	Did anyone ever try to put their penis into your mouth before you were 16?	8,758 (1.8)	0.803	
Non-sexual abuse	A parent was emotionally cruel to you before you were 17	8,984 (8.0)	0.940	RMSEA 0.060 CFI 0.991 TLI 0.972
	A parent was physically cruel to you before you were 17	8,984 (3.4)	0.900	
	Did you feel neglected emotionally during your childhood?	7,198 (21.4)	0.781	
	Were you physically neglected as a child (e.g. not fed or clothed properly)?	7,203 (1.7)	0.738	
Maladaptive family functioning	Home stability (RS)[a]	8,959 (12.1)	0.771	RMSEA 0.062 CFI 0.961 TLI 0.952
	Did your parents have serious arguments?	8,984 (27.7)	0.788	
	Was your parent’s relationship violent?	6,696 (13.3)	0.893	
	Was your parent’s relationship affectionate? (RS)	6,797 (12.4)	0.782	
	Was your parent’s relationship quarrelsome?	6,832 (77.8)	0.629	
	Was your parent’s relationship happy? (RS)	6,833 (4.9)	0.905	
	Was your parent’s relationship frightening?	6,756 (19.2)	0.869	
	Was your parent’s relationship friendly? (RS)	6,802 (3.6)	0.910	
	Was your parent’s relationship respectful? (RS)	6,776 (8.5)	0.884	
	Was your parent’s relationship remote or distant?	6,747 (42.5)	0.757	
	Before age 17 years were your parents separated or divorced?	8,984 (16.3)	0.700	
	Any paternal absence before 11 years of age	8,984 (12.7)	0.462	
Parental mental illness	Was either parent mentally ill before age 17 years?	8,984 (4.6)	0.711	RMSEA 0.048 CFI 0.967 TLI 0.900
	Did your mother have depression or nerves?	8,657 (22.5)	0.600	
	Did your mother have an alcohol problem?	8,900 (2.7)	0.811	
	Did your father have an alcohol problem?	8,717 (6.5)	0.387	

Maternal lack of care	Did your mother speak to you in a warm and friendly voice? (RS)	8,938 (20.3)	0.877	RMSEA 0.062<br>CFI 0.984<br>TLI 0.980
	Did your mother help you as much as you needed? (RS)	8,954 (19.3)	0.902	
	Did your mother seem emotionally cold to you?	8,945 (4.9)	0.755	
	Problems understood by the mother (RS)	8,966 (49.1)	0.838	
	Was your mother affectionate towards you? (RS)	8,967 (29.1)	0.877	
	Did your mother make you feel you were not wanted?	8,973 (3.6)	0.640	
	Did your mother talk things over with you? (RS)	8,979 (55.2)	0.829	
	Did your mother praise you? (RS)	8,959 (47.2)	0.816	
	Did your mother enjoy talking things over with you? (RS)	8,858 (19.5)	0.897	
	Did your mother frequently smile at you? (RS)	8,913 (10.5)	0.892	
	Did your mother seem to understand what you needed or wanted? (RS)	8,859 (22.3)	0.907	
	Did your mother make you feel better when you were upset? (RS)	8,892 (13.3)	0.890	
Maternal overprotection	Did your mother allow you to do things you liked doing? (RS)	8,976 (41.5)	0.662	RMSEA 0.082<br>CFI 0.953<br>TLI 0.930
	Did your mother try to control what you did?	8,966 (25.2)	0.507	
	Did your mother let you decide things for yourself? (RS)	8,981 (54.1)	0.717	
	Did your mother give you the freedom you wanted? (RS)	8,973 (63.9)	0.851	
	Did your mother let you go out as often as you wanted? (RS)	8,915 (54.7)	0.775	
	Was your mother overprotective of you?	8,934 (21.0)	0.491	
	Did your mother allow you to dress in any way you pleased? (RS)	8,919 (42.8)	0.665	
Second order factor analysis of cumulative psychosocial adversity	Sexual abuse	NA	0.442	RMSEA 0.040<br>CFI 0.935<br>TLI 0.931
	Non-sexual abuse	NA	0.975	
	Maladaptive family functioning	NA	0.706	
	Parental mental illness	NA	0.704	
	Lack of care	NA	0.753	
	Overprotection	NA	0.552	

RS: reversed scale

[a] Home stability was defined based on a general question of self-evaluation of whether the childhood home was very stable, fairly stable, unstable or very unstable.

Table S2 Distribution of background characteristics among included and excluded individuals

Characteristics	Women with information needed to be included in analysis of age at menarche		
	Yes (n= 8,984)	No (n= 5,557)	p-values
Age at delivery/enrolment, Mean(SD)	28.5 (4.8)	27.1 (5.2)	<0.001
Missing, N(%)	0	645	
Ethnicity, N(%)			<0.001
White/European	8,798 (97.9)	3,129 (95.9)	
Other	186 (2.1)	135 (4.1)	
Missing	0	2,293	
Education, N(%)			<0.001
A level or above	3,499 (39.0)	859 (25.6)	
O level or below	5,485 (61.0)	2,497 (74.4)	
Missing	0	2,201	
Age at menarche, Mean(SD)	12.9 (1.5)	12.8 (1.6)	0.016
Missing, N(%)	0	3,436	

Percentages are based on observed values to facilitate comparability.

Table S3 Associations between the latent constructs from the first order factor analysis

Latent construct	Maladaptive family functioning	Parental mental illness	Lack of care	Maternal overprotection	Sexual abuse	Non-sexual abuse
Maladaptive family functioning	1					
Parental mental illness	0.706	1				
Lack of care	0.501	0.447	1			
Maternal overprotection	0.207	0.185	0.558	1		
Sexual abuse	0.378	0.404	0.281	0.159	1	
Non-sexual abuse	0.804	0.681	0.679	0.363	0.513	1

The coefficients reflect the change in the standard deviation in one factor per standard deviation increase in the other factor

Table S4 Associations of childhood psychosocial adversity with age at menarche: sensitivity analysis restricted to individuals with direct information on childhood socio-economic position

(n=5,389)

Exposure	Unadjusted		Adjusted	
	Mean difference in years (95% CI)	p-value	Mean difference in years (95% CI)	p-value
Total psychosocial adversity	-0.013 (-0.062, 0.036)	0.601	-0.015 (-0.064, 0.034)	0.540
Lack of care	0.006 (-0.039, 0.051)	0.784	-0.001 (-0.046, 0.044)	0.982
Maladaptive family functioning	0.003 (-0.046, 0.052)	0.913	0.012 (-0.037, 0.061)	0.631
Nonsexual abuse	-0.018 (-0.081, 0.045)	0.560	-0.016 (-0.077, 0.045)	0.604
Overprotective parenting	-0.024 (-0.069, 0.021)	0.301	-0.033 (-0.078, 0.012)	0.160
Parental mental illness	-0.019 (-0.080, 0.042)	0.548	0.001 (-0.062, 0.064)	0.969
Sexual abuse	-0.165 (-0.230, -0.100)	<0.001	-0.158 (-0.223, -0.093)	<0.001

Beta coefficients are interpreted as a mean difference in age at menarche in years per standard deviation increase in psychosocial adversity.

Adjusted for age at recruitment, ethnicity, paternal occupational status and parental highest educational qualifications.

Table S5 Associations of childhood psychosocial adversity with age at menopause: sensitivity analysis restricted to individuals with direct information on childhood socio-economic position

(n=626)

Exposure	Unadjusted		Adjusted	
	Mean difference in years (95% CI)	p-value	Mean difference in years (95% CI)	p-value
Total psychosocial adversity	0.082 (-0.269, 0.433)	0.644	0.046 (-0.266, 0.358)	0.771
Lack of care	0.127 (-0.204, 0.458)	0.452	0.070 (-0.214, 0.354)	0.627
Maladaptive family functioning	0.018 (-0.327, 0.363)	0.918	0.064 (-0.252, 0.380)	0.693
Nonsexual abuse	0.027 (-0.406, 0.460)	0.904	-0.037 (-0.409, 0.335)	0.845
Overprotective parenting	0.012 (-0.323, 0.347)	0.946	-0.055 (-0.361, 0.251)	0.726
Parental mental illness	-0.127 (-0.582, 0.328)	0.585	-0.018 (-0.408, 0.372)	0.928
Sexual abuse	-0.054 (-0.546, 0.438)	0.830	-0.116 (-0.579, 0.347)	0.622

Beta coefficients are interpreted as a mean difference in age at menopause in years per standard deviation increase in psychosocial adversity.

Adjusted for age at recruitment, ethnicity, paternal occupational status and parental highest educational qualifications.

Table S6 Associations of childhood psychosocial adversity with length of reproductive lifespan: sensitivity analysis restricted to individuals with direct information on childhood socio-economic position

(n=557)

Exposure	Unadjusted		Adjusted	
	Mean difference in years (95% CI)	p-value	Mean difference in years (95% CI)	p-value
Total psychosocial adversity	-0.046 (-0.434, 0.342)	0.816	-0.098 (-0.437, 0.241)	0.572
Lack of care	-0.040 (-0.403, 0.323)	0.829	-0.104 (-0.408, 0.200)	0.501
Maladaptive family functioning	0.049 (-0.330, 0.427)	0.798	0.082 (-0.255, 0.419)	0.633
Nonsexual abuse	-0.064 (-0.499, 0.371)	0.774	-0.145 (-0.513, 0.223)	0.438
Overprotective parenting	-0.055 (-0.429, 0.319)	0.773	-0.154 (-0.499, 0.191)	0.380
Parental mental illness	0.053 (-0.441, 0.547)	0.832	0.105 (-0.324, 0.534)	0.631
Sexual abuse	-0.157 (-0.676, 0.362)	0.552	-0.210 (-0.694, 0.274)	0.394

Beta coefficients are interpreted as a mean difference in length of reproductive lifespan in years per standard deviation increase in psychosocial adversity.

Adjusted for age at recruitment, ethnicity, paternal occupational status and parental highest educational qualifications.

Table S7 Associations of childhood psychosocial adversity with age at menarche: sensitivity analysis using a complete case analysis for all covariates used to inform the latent constructs in first order factors

(n=5,568)

Exposure	Model 1		Model 2		Model 3	
	Mean difference in years (95% CI)	p-value	Mean difference in years (95% CI)	p-value	Mean difference in years (95% CI)	p-value
Total psychosocial adversity	0.013 (-0.036, 0.062)		0.014 (-0.035, 0.063)		0.014 (-0.035, 0.063)	
Lack of care	0.031 (-0.014, 0.076)	0.167	0.031 (-0.014, 0.076)	0.179	0.030 (-0.015, 0.075)	0.192
Maladaptive family functioning	0.012 (-0.035, 0.059)	0.611	0.017 (-0.030, 0.064)	0.472	0.017 (-0.030, 0.064)	0.468
Nonsexual abuse	-0.005 (-0.066, 0.056)	0.874	-0.003 (-0.064, 0.058)	0.925	-0.002 (-0.063, 0.059)	0.958
Overprotective parenting	0.000 (-0.045, 0.045)	0.983	-0.003 (-0.048, 0.042)	0.891	-0.004 (-0.049, 0.041)	0.860
Parental mental illness	-0.021 (-0.084, 0.042)	0.503	-0.022 (-0.083, 0.039)	0.480	-0.022 (-0.083, 0.039)	0.480
Sexual abuse	-0.169 (-0.238, -0.100)	<0.001	-0.164 (-0.233, -0.095)	<0.001	-0.164 (-0.233, -0.095)	<0.001

Beta coefficients are interpreted as a mean difference in age at menarche in years per standard deviation increase in psychosocial adversity.

Model 1 Unadjusted.

Model 2 Adjusted for age at recruitment and ethnicity.

Model 3 Adjusted for age at recruitment, educational qualifications and ethnicity.

Table S8 Associations of childhood psychosocial adversity with age at menopause: sensitivity analysis using a complete case analysis for all covariates used to inform the latent constructs in first order factors

(n=709)

Exposure	Model 1		Model 2		Model 3	
	Mean difference in years (95% CI)	p-value	Mean difference in years (95% CI)	p-value	Mean difference in years (95% CI)	p-value
Total psychosocial adversity	0.094 (-0.237, 0.425)	0.576	0.092 (-0.194, 0.378)	0.529	0.075 (-0.209, 0.360)	0.602
Lack of care	0.131 (-0.181, 0.443)	0.411	0.120 (-0.152, 0.392)	0.389	0.109 (-0.161, 0.380)	0.430
Maladaptive family functioning	-0.030 (-0.352,0.291)	0.851	0.057 (-0.227, 0.341)	0.696	0.056 (-0.228, 0.340)	0.701
Nonsexual abuse	0.030 (-0.464, 0.524)	0.851	0.002 (-0.323, 0.327)	0.990	-0.041 (-0.353, 0.271)	0.798
Overprotective parenting	0.108 (-0.202, 0.418)	0.494	0.020 (-0.262, 0.302)	0.888	0.007 (-0.273, 0.002)	0.963
Parental mental illness	-0.179 (-0.644, 0.286)	0.450	-0.057 (-0.484, 0.370)	0.795	-0.082 (-0.513, 0.349)	0.707
Sexual abuse	0.220 (-0.292, 0.732)	0.397	0.262 (-0.240, 0.764)	0.306	0.258 (-0.230, 0.746)	0.300

Beta coefficients are interpreted as a mean difference in age at menopause in years per standard deviation increase in psychosocial adversity.

Model 1 Unadjusted.

Model 2 Adjusted for age at recruitment and ethnicity.

Model 3 Adjusted for age at recruitment, educational qualifications and ethnicity.

Table S9 Associations of childhood psychosocial adversity with length of reproductive lifespan: sensitivity analysis using a complete case analysis for all covariates used to inform the latent constructs in first order factors

(n=632)

Exposure	Model 1		Model 2		Model 3	
	Mean difference in years (95% CI)	p-value	Mean difference in years (95% CI)	p-value	Mean difference in years (95% CI)	p-value
Total psychosocial adversity	0.049 (-0.318, 0.416)	0.794	0.056 (-0.265, 0.377)	0.738	0.032 (-0.286, 0.350)	0.845
Lack of care	0.016 (-0.325, 0.357)	0.926	0.007 (-0.290, 0.305)	0.961	-0.009 (-0.303, 0.285)	0.954
Maladaptive family functioning	0.047 (-0.318, 0.412)	0.799	0.156 (-0.165, 0.477)	0.342	0.155 (-0.164, 0.474)	0.342
Nonsexual abuse	0.030 (-0.464, 0.524)	0.905	-0.050 (-0.413, 0.313)	0.786	-0.102 (-0.453, 0.249)	0.566
Overprotective parenting	0.110 (-0.243, 0.463)	0.542	0.005 (-0.326, 0.336)	0.977	-0.019 (-0.348, 0.310)	0.911
Parental mental illness	-0.070 (-0.570, 0.430)	0.785	0.122 (-0.364, 0.608)	0.622	0.114 (-0.370, 0.598)	0.643
Sexual abuse	0.275 (-0.280, 0.830)	0.331	0.354 (-0.216, 0.924)	0.223	0.347 (-0.206, 0.900)	0.218

Beta coefficients are interpreted as a mean difference in length of reproductive lifespan in years per standard deviation increase in psychosocial adversity.

Model 1 Unadjusted.

Model 2 Adjusted for age at recruitment and ethnicity.

Model 3 Adjusted for age at recruitment, educational qualifications and ethnicity.

Table S10 Associations of childhood psychosocial adversity with age at menarche: sensitivity analysis including all individuals with information on at least one measure of childhood adversity

(n=10,372)

Exposure	Model 1		Model 2		Model 3	
	Mean difference in years (95% CI)	p-value	Mean difference in years (95% CI)	p-value	Mean difference in years (95% CI)	p-value
Total psychosocial adversity	0.006 (-0.031, 0.043)	0.734	0.007 (-0.028, 0.042)	0.712	0.006 (-0.029, 0.041)	0.765
Lack of care	0.032 (-0.001, 0.065)	0.062	0.027 (-0.006, 0.060)	0.106	0.026 (-0.007, 0.059)	0.124
Maladaptive family functioning	0.012 (-0.025, 0.049)	0.538	0.022 (-0.015, 0.059)	0.253	0.021 (-0.016, 0.058)	0.270
Nonsexual abuse	-0.020 (-0.065, 0.025)	0.371	-0.019 (-0.064, 0.026)	0.413	-0.018 (-0.063, 0.027)	0.431
Overprotective parenting	-0.001 (-0.034, 0.032)	0.955	-0.008 (-0.041, 0.025)	0.664	-0.009 (-0.042, 0.024)	0.624
Parental mental illness	-0.030 (-0.075, 0.015)	0.199	-0.032 (-0.077, 0.013)	0.166	-0.032 (-0.077, 0.013)	0.166
Sexual abuse	-0.184 (-0.235, -0.133)	<0.001	-0.175 (-0.226, -0.124)	<0.001	-0.175 (-0.226, -0.124)	<0.001

Beta coefficients are interpreted as a mean difference in age at menarche in years per standard deviation increase in psychosocial adversity.

Model 1 Unadjusted.

Model 2 Adjusted for age at recruitment and ethnicity.

Model 3 Adjusted for age at recruitment, educational qualifications and ethnicity.

Table S11 Associations of childhood psychosocial adversity with age at menopause: sensitivity analysis including all individuals with information on at least one measure of childhood adversity

(n=1,076)

Exposure	Model 1		Model 2		Model 3	
	Mean difference in years (95% CI)	p-value	Mean difference in years (95% CI)	p-value	Mean difference in years (95% CI)	p-value
Total psychosocial adversity	0.057 (-0.221, 0.335)	0.691	0.050 (-0.203, 0.303)	0.696	0.040 (-0.209, 0.289)	0.755
Lack of care	0.122 (-0.136, 0.381)	0.357	0.105 (-0.126, 0.336)	0.374	0.094 (-0.135, 0.323)	0.424
Maladaptive family functioning	-0.075 (-0.355, 0.205)	0.599	0.009 (-0.242, 0.260)	0.945	0.014 (-0.237, 0.265)	0.910
Nonsexual abuse	0.046 (-0.326, 0.418)	0.808	0.049 (-0.282, 0.380)	0.771	0.034 (-0.282, 0.350)	0.831
Overprotective parenting	0.083 (-0.189, 0.355)	0.551	-0.060 (-0.309, 0.189)	0.637	-0.072 (-0.319, 0.175)	0.571
Parental mental illness	-0.161 (-0.543, 0.221)	0.407	-0.053 (-0.382, 0.276)	0.754	-0.082 (-0.413, 0.249)	0.630
Sexual abuse	-0.147 (-0.537, 0.243)	0.460	-0.126 (-0.473, 0.221)	0.476	-0.134 ((-0.479, 0.211)	0.446

Beta coefficients are interpreted as a mean difference in age at menopause in years per standard deviation increase in psychosocial adversity.

Model 1 Unadjusted.

Model 2 Adjusted for age at recruitment and ethnicity.

Model 3 Adjusted for age at recruitment, educational qualifications and ethnicity.

Table S12 Associations of childhood psychosocial adversity with length of reproductive lifespan: sensitivity analysis including all individuals with information on at least one measure of childhood adversity

(n=945)

Exposure	Model 1		Model 2		Model 3	
	Mean difference in years (95% CI)	p-value	Mean difference in years (95% CI)	p-value	Mean difference in years (95% CI)	p-value
Total psychosocial adversity	0.024 (-0.284, 0.332)	0.878	0.018 (-0.264, 0.300)	0.900	0.005 (-0.273, 0.283)	0.974
Lack of care	0.022 (-0.260, 0.304)	0.877	-0.004 (-0.259, 0.251)	0.977	-0.022 (-0.275, 0.231)	0.862
Maladaptive family functioning	0.006 (-0.304, 0.316)	0.969	0.119 (-0.161, 0.399)	0.405	0.131 (-0.149, 0.411)	0.360
Nonsexual abuse	-0.033 (-0.331, 0.265)	0.827	-0.006 (-0.355, 0.343)	0.973	-0.007 (-0.342, 0.328)	0.965
Overprotective parenting	0.061 (-0.241, 0.363)	0.694	-0.116 (-0.398, 0.166)	0.419	-0.138 (-0.418, 0.142)	0.333
Parental mental illness	-0.017 (-0.442, 0.408)	0.938	0.071 (-0.315, 0.457)	0.719	0.040 (-0.342, 0.422)	0.836
Sexual abuse	-0.006 (-0.429, 0.417)	0.980	0.044 (-0.348, 0.436)	0.828	0.023 (-0.183, 0.229)	0.903

Beta coefficients are interpreted as a mean difference in length of reproductive lifespan in years per standard deviation increase in psychosocial adversity.

Model 1 Unadjusted.

Model 2 Adjusted for age at recruitment and ethnicity.

Model 3 Adjusted for age at recruitment, educational qualifications and ethnicity.

Table S13 Associations of childhood psychosocial adversity with age at menarche: sensitivity analysis in the sample used to analyse length of reproductive lifespan

(n=841)

Exposure	Model 1		Model 2		Model 3	
	Mean difference in years (95% CI)	p-value	Mean difference in years (95% CI)	p-value	Mean difference in years (95% CI)	p-value
Total psychosocial adversity	0.043 (-0.079, 0.165)	0.485	0.042 (-0.078, 0.162)	0.494	0.039 (-0.081, 0.159)	0.527
Lack of care	0.085 (-0.027, 0.197)	0.141	0.085 (-0.025, 0.195)	0.132	0.084 (-0.026, 0.194)	0.135
Maladaptive family functioning	-0.059 (-0.188, 0.070)	0.367	-0.062 (-0.189, 0.065)	0.336	-0.062 (-0.187, 0.063)	0.340
Nonsexual abuse	0.001 (-0.146, 0.148)	0.993	0.000 (-0.145, 0.145)	1.000	-0.002 (-0.147, 0.143)	0.977
Overprotective parenting	0.081 (-0.031, 0.193)	0.157	0.081 (-0.031, 0.193)	0.158	0.081 (-0.031, 0.193)	0.154
Parental mental illness	-0.096 (-0.255, 0.063)	0.234	-0.095 (-0.252, 0.062)	0.234	-0.114 (-0.261, 0.033)	0.128
Sexual abuse	-0.083 (-0.248, 0.082)	0.325	-0.075 (-0.232, 0.082)	0.345	-0.086 (-0.237, 0.065)	0.265

Beta coefficients are interpreted as a mean difference in age at menarche in years per standard deviation increase in psychosocial adversity.

Model 1 Unadjusted.

Model 2 Adjusted for age at recruitment and ethnicity.

Model 3 Adjusted for age at recruitment, educational qualifications and ethnicity.

Table S14 Associations of biological father absence and age at menarche, age at menopause and length of reproductive lifespan

Outcome	Model	None	Absent by 5 years of age<br>Mean difference in years (95% CI)	Absent first between 6-11 years of age<br>Mean difference in years (95% CI)
Age at menarche in years (n=8,984)	Model 1	Ref	0.026 (-0.101, 0.153)	-0.111 (-0.241, 0.018)
	Model 2	Ref	0 .078 (-0.051, 0.207)	-0.061 (-0.192, 0.070)
	Model 3	Ref	0.067 (-0.062, 0.196)	-0.066 (-0.196, 0.065)
Age at menopause in years (n=945)	Model 1	Ref	-1.415 (-2.671, -0.158)	-1.881 (-3.380, -0.382)
	Model 2	Ref	-0.745 (-1.888, 0.398)	-1.791 (-3.144, -0.438)
	Model 3	Ref	-0.694 (-1.837, 0.449)	-1.733 (-3.086, -0.380)
Length of reproductive lifespan in years (n=841)	Model 1	Ref	-1.228 (-2.686, 0.230)	-1.310 (-3.004, 0.384)
	Model 2	Ref	-0.880 (-2.221, 0.460)	-1.002 (-2.551, 0.546)
	Model 3	Ref	-0.768 (-2.110, 0.573)	-0.884 (-2.433, 0.665)

Model 1 Unadjusted.

Model 2 Adjusted for age at recruitment and ethnicity.

Model 3 Adjusted for age at recruitment, educational qualifications and ethnicity.

Table S15 Associations between paternal absence the first 11 years of life and the other latent constructs for childhood psychosocial adversity from the first order factor analysis

Outcome	Mean difference in standard deviation (95% CI)	p-value
Lack of care	0.269 (0.198, 0.340)	<0.001
Nonsexual abuse	0.636 (0.554, 0.718)	<0.001
Overprotective parenting	0.121 (0.048, 0.009)	0.001
Parental mental illness	0.451 (0.355, 0.547)	<0.001
Sexual abuse	0.509 (0.417, 0.601)	<0.001

Beta coefficients are interpreted as the difference in standard deviation of the latent construct for psychosocial adversity between those who experienced paternal absence the first 11 years of life compared to those who did not experience paternal absence.

Figure S1 A figure illustrating the second order factor analysis of childhood psychosocial adversity in relation to female reproductive timing

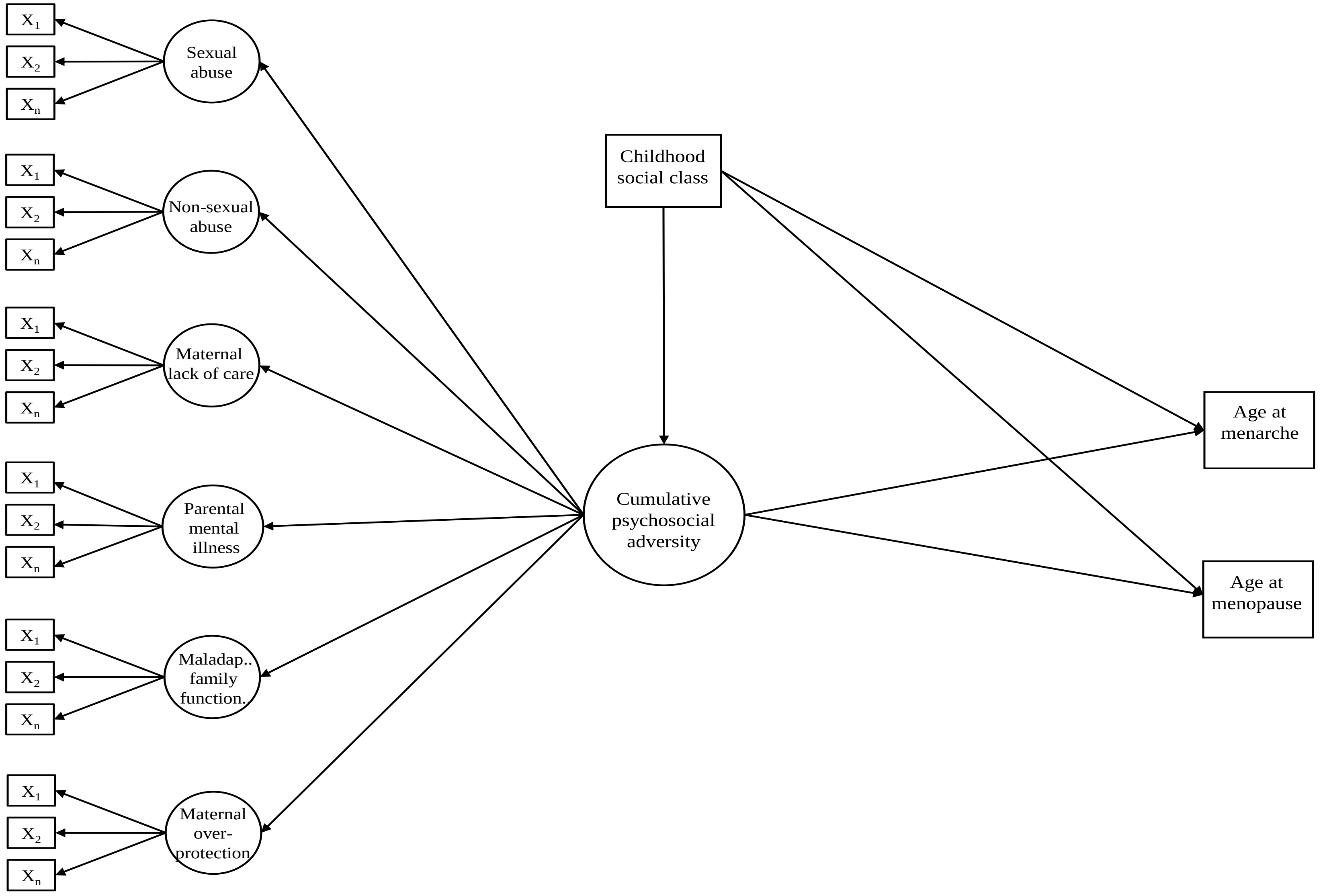

Supplement: Supplementary file 1 [file jech-2017-209488supp001.pdf]
